# The effects of nationality differences and work stressors on work adjustment for foreign nurse aides

**DOI:** 10.1186/1472-6963-11-192

**Published:** 2011-08-17

**Authors:** Fen Fen Huang, Hsieh Hua Yang

**Affiliations:** 1 Department of Health Care Administration, Oriental Institute of Technology, Taiwan, 58, Sec. 2, Szechwan Rd., Banciao Dist, New Taipei City 220, Taiwan

**Keywords:** Work Stressors, Work Adjustment, Foreign Nurse Aides

## Abstract

**Background:**

The main purpose of this study was to discuss the nationality differences of foreign nurse aides and the effect of work stressors influencing work adjustment. And of helping them adapt to Taiwanese society, we summarized the difficulties that foreign nurse aides face in Taiwan.

**Methods:**

The subjects included 80 foreign nurse aides from the Philippines, Indonesia, and Vietnam who worked in long-term care facilities in Tao Yuan County. We recruited volunteers at the participating facilities to complete the anonymous questionnaires. The return rate of the questionnaire was 88.75%. The validated instruments of Hershenson's (1981) and Schaefer and Moos (1993) were adopted to measure work stressors and work adjustment, respectively. A forward-backward translation process was used in this study.

**Results:**

Indonesian foreign nurse aides respect their work, and are better workers than Vietnamese and Filipino nurse aids in many respects, which shows how the nationality of the foreign nurse aides might affect work adjustment. The stress created from patient care tasks influenced the foreign nurse aides' personal relationships at work and also affected their attitude when they performed their tasks. In addition, pressure from their supervisors might have affected their work skills, work habits, personal relationships, self-concepts or work attitudes. Moreover, a heavy workload and improper scheduling might have affected the personal relationships and work attitudes of the foreign nurse aides. It was found that work stressors had a significant correlation with work adjustment.

**Conclusions:**

The results of the present study indicate that training programs are important factors for work adjustment among foreign nurse aides. Furthermore, celebration and leisure activities could be provided to release them from work stressors. More effort should be put into improving the working environment, namely providing a more supportive and enriching atmosphere. Based on these findings, we have a better understanding of how to assist foreign nurse aides in the future.

## Background

Since the National Health Insurance was implemented in Taiwan, the average life-span has increased. According to the report by the Ministry of the Interior, the population over the age of 65 will have grown from 10% of the total population in 2006 to 13% in 2014, and it will only continue growing to 37% in 2051 [1]. Many elderly people have to live in long-term care facilities and depend on nurse aides to provide their direct care. Moreover, the population of people over the age of 75 will increase from 950,000 people in 2006 to 1,260,000 people in 2014 and 3,690,000 people in 2051. The statistics of Taiwan and other countries are shown in the following table 1[2]:

**Table 1:** The Ratio Of Aging Population (%)

	1995	1996	1997	1998	1999	2000	2001	2002	2003	2004	1995-2004
Taiwan	7.6	7.9	8.1	8.3	8.4	8.6	8.8	9.0	9.2	9.5	1.9
Japan	14.5	15.1	15.7	16.2	16.7	17.3	18.0	18.5	19.0	19.5	5.0
Korea	5.9	6.1	6.4	6.6	6.9	7.2	7.6	7.9	8.3	8.7	2.8
England	15.8	15.9	15.9	15.9	15.8	15.8	15.9	15.9	16.0	16.0	0.2
France	15.2	15.4	15.6	15.8	15.9	16.1	16.2	16.2	16.3	16.3	1.1
Germany	16.1	16.3	16.5	16.6	16.8	17.2	17.6	18.1	18.6	19.3	3.2
American	12.7	12.7	12.6	12.5	12.5	12.4	12.4	12.4	12.4	12.4	-0.3

The Ratio Of Aging Population = (Population Over 65 years/Total Population)*100.

With the increasing elderly population comes a change in family structure. The younger population will no longer be able to care for their elderly parents. As a result, they will have to employ caregivers or send their parents to long-term care facilities [3]. Since 1992, foreign laborers have started immigrating to Taiwan, including 167,980 foreigners who became foreign nurse aides and caregivers [4]. Foreign nurse aides have become an important human resource in long-term care facilities. However, the differences of language, living habits, and cultures might cause some problems and friction between the foreign nurse aides and the Taiwanese elderly, which affects the quality of care [5,6] and, subsequently, the work adjustment for foreign nurse aids [7]. In previous study, it was mentioned that the barriers to quality of care could be language and communication inadequacy as well as cultural differences [8]. Obviously, the work adjustment of foreign laborers is the most important issue. It is important to explore the effects of nationality differences and work stressors on work adjustment. The purpose of this study was to explore the relationships between nationality, work stressors, and work adjustment.

Work adjustment describes how easily someone can adapt to their working environment, which might include factors such as work content, relationship with co-workers, management style of the boss, adaptation to environment differences, working compatibility, and working regulations. When discussing work adjustment, Parsons' trait-and-factor theory is the most adopted theory [9]. Other important theories include the model of Hershenson's work adjustment and the theory of work compatibility by Dawis and Lofquist [10]. Work adjustment in this study was based on the theory by Hershenson, which describes how people's working values can influence the atmosphere in a welfare institution. Work satisfaction is the internal indicator while the observation from a supervisor and a Taiwanese co-worker is the standard of external satisfaction. Work adjustment is achieved when both internal and external indicators are satisfied.

Recent studies have shown that work stressors are not limited to the work environment, and that a worker's personal situation might contribute to any physical, psychological, or behavioral changes in their life, i.e. dealing with interpersonal conflicts [5,11-13], lack of support [14-16], and inequality of opportunities [17,18]. Lazarus and Folkman concluded that stress resulted from environmental events or stressors [19]. The amount of daily conflict experienced by an individual appeared to be the most important environmental event in determining adaptation and health [19].

Therefore, the aims of the present study were (1) to discover the relationship between nationality differences and work adjustment and (2) to investigate the foreign nurse aides' ratings on the work stressors and work adjustment. We wanted to find a solution to the problem that foreign nurse aides have, to provide appropriate support to help them adapt to Taiwanese society, and to allow foreign nurse aides to gain work dignity in Taiwan. In this study, two research questions were proposed as discussed below.

### Research Question 1

Is there any difference in work adjustment between nurse aides of different nationalities?

Most of the foreign nurse aides are from Vietnam, the Philippines, or Indonesia. Culture is a set of guidelines that individuals inherit as members of a particular society. The context of culture comprises historical, economic, social, political, and geographical elements [8]. Thus, different cultural backgrounds of people from different countries might cause them to think, experience life, and behave differently [7]. As a result, working in another country is an important event for foreign nurse aides, and it can alter their life dramatically [6]. Past researchers found that nationality has a significant influence on work adjustment [20]. Because of the different customs and cultures and different ways of thinking, workers from different nations will experience working in a new environment in different ways.

### Research Question 2

Is there any correlation between work stressors and work adjustment for foreign nurse aides?

Care-providers were interviewed, and they informed the researchers that they could not satisfy patients when performing their tasks due to their assigned heavy workload, strict rules set by human resources, and lack of resources [21-23]. In previous articles, scholars have discussed work stressors and adjustments in many other aspects, including self-esteem [24,25], racism [15,26], trusts in management [27] and self-efficacy [28,6].

**Figure 1:**
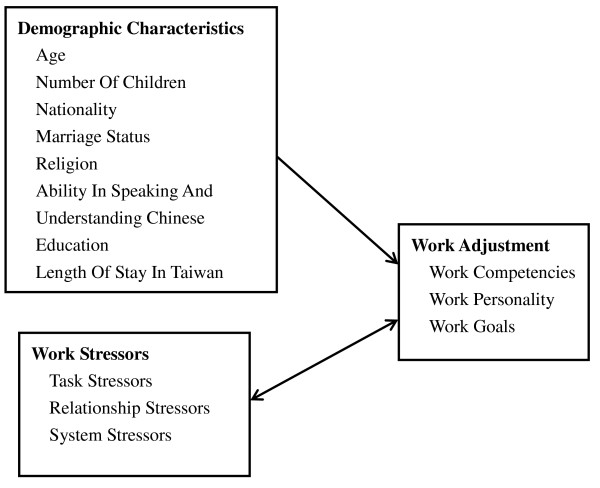
**Research Framework**.

The framework of this research is as follows (Figure 1). Nationality differences and work stressors are key influencing factors for work adjustment. The research framework included three components:

(1) Demographic profile, including age, marital status and nationality, of the foreign nurse aides.

(2) Work stressors, including tasks, relationships and system stressors.

(3) Work adjustment, including work competence, personality and goals.

## Methods

### Participants And Setting

The participants were foreign nurse aides from the Philippines, Indonesia, and Vietnam who were invited from long-term care facilities in Tao Yuan County in Taiwan. This research was performed with the approval of an appropriate ethics committee (IRB, Institutional/Independent Review Board) from the long-term care facilities. A forward-backward translation process was used. We invited the translators from the long-term care facilities' human resource departments to participate in translating the questionnaire into three different languages. The questionnaires were translated from English to Vietnamese and Indonesian, and then translated back into English by other translators. Both English versions were reviewed to make sure the contents were the same.

Twenty-four long-term care facilities were contacted. We interviewed by phone each of the facilities that were willing to participate in the research; 11 out of the 24 participated. We recruited volunteers at the participating facilities to complete the anonymous questionnaires. Eighty foreign nurse aides were invited, and 71 completed the questionnaires. The return rate of the questionnaire was 88.75%.

### Measurement

Work Adjustment Questionnaire

Two instruments were adopted to measure work stressors and work adjustment. To measure work adjustment, we used the Hershenson method (1981), but revised the descriptions of work environment and the target term to foreign nurse aide to make it fit our research [29]. The concept of Hershenson's model is illustrated as follows: "The model posits that work adjustment involves the development of three domains: Work personality (i.e., self-concept as a worker and a personal system of motivation for work), work competencies (i.e., work habits, physical and mental skills applicable to jobs, and work-related interpersonal skills), and appropriate, crystallized work goals" (Hershenson, 1981:92). There questionnaire has 20 questions and measures the personal conditions of work adjustment, including:

1. Work competencies: the situation of foreign nurse aides regarding work performance.

2. Work personality: foreign nurse aides' self-concepts as workers and their motivation for work.

3. Work goals: the target that would make foreign nurse aides satisfied spiritually.

According to the present study, the Cronbach's α for all questions is 0.89, and the Cronbach's α for coincidence of construct are 0.81, 0.79, and 0.77. The average is more than 0.7, which means the questionnaire has good reliability.

Work Stressors Inventory (WSI)

The questions in this section refer to the WSI of Schaefer and Moos (1993) [30]. Again, we invited researchers and professionals to verify the questions considering the purpose of the research and to revise some of the questions and translate them into three different languages. There are 23 questions for measuring the feeling of work stressors, including:

1. Task stressors: the opinions and feelings of the foreign nurse aides about their work.

2. Relationship stressors: the situation of whether the foreign nurse aides are getting along with their boss and/or co-workers.

3. System stressors: the opinions of the foreign nurse aides about the work environment of the facilities.

**Figure 2:**
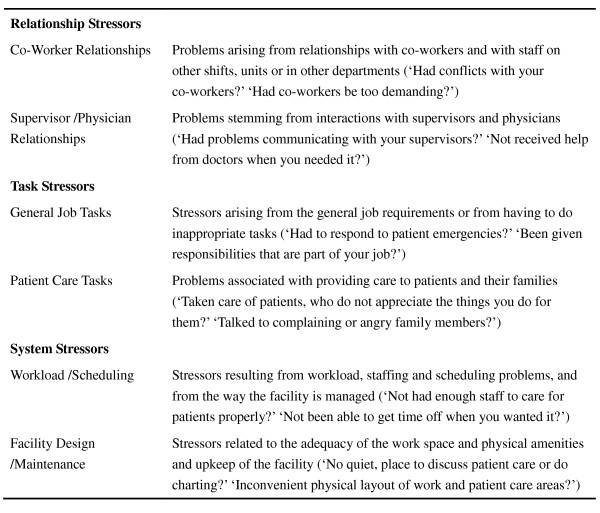
**Work Stressors Inventory (WSI) Subscale Descriptions And Item Examples**.

According to the present study, the Cronbach's α of all questions is 0.85, and the Cronbach's α of coincidence of construct are 0.76, 0.72, and 0.71. The average is more than 0.7, which means the questionnaire has good reliability. Schaefer and Moos illustrated the WSI subscale descriptions and item examples as Figure 2 (Schaefer and Moos, 1993:290).

### Statistical Data Analysis

One-way ANOVA was used for inferential statistics. This analysis was focused on discussing the influence of work adjustment on foreign nurse aides with nationality differences. Bivariate Pearson-product moment correlation was used to verify the correlation coefficient and significance between work stressors and work adjustment. Its focus was to discuss the association between work stressors and work adjustment of the foreign nurse aides. All p-values given were two-tailed. A p-value of less than .05 was considered significant [31]. Values are given as the mean and standard deviation. Data were calculated using the SPSS 17.0 software package.

## Results

### Demographic Characteristics Of Participants

The ages of the foreign nurse aides were mostly 20 to 25 years old (44.3%). The nurses were predominately Vietnamese (42.3%). The percentage of foreign nurse aides with children was 54.3%. Regarding religion, 53.5% of the sample foreign nurse aides are Muslim. The percentage of married foreign nurse aids is 45.7%. Regarding language, 50.7% can speak basic Chinese. Regarding education, 67.6% graduated from high school. Regarding length of stay in Taiwan, about 63.2% of them are working their first year in Taiwan (Table 2).

**Table 2:** Demographic Characteristic Of Participants

Variable		Number	Percentage
Age	20-25 years old	31	44.3%
	26-30 years old	18	25.7%
	31-35 years old	12	17.1%
	36-40 years old	8	11.4%
	Over 41 years old	1	1.4%
Number Of Children	None	32	45.7%
	One	24	34.3%
	Two	12	17.1%
	Three	1	1.4%
	More than four	1	1.4%
Nationality	Philippine	17	23.9%
	Vietnam	30	42.3%
	Indonesia	24	33.8%
Marriage Status	Married	32	45.7%
	Unmarried	20	28.6%
	Divorce or live apart	5	7.1%
	Widowed	1	1.4%
	Other	12	17.1%
Religion	Catholicism	17	23.9%
	Christianity	5	7.0%
	Buddhism	6	8.5%
	Islam	38	53.5%
	Other	5	7.0%
Ability In Speaking And Understanding Chinese	Fluent	12	16.9%
	Above average	24	33.8%
	Average	16	22.5%
	Poor	18	25.4%
	None	1	1.4%
Education	0-5 years	13	19.1%
	6-10 years	9	13.2%
	11-15 years	46	67.6%
Length Of Stay In Taiwan	0-1 year	43	63.2%
	1-2 years	12	17.6%
	2-3 years	4	5.9%
	3-4 years	6	8.8%
	Over 4 years	3	4.4%

### Work Stressors And Work Adjustment

This study was used one-way ANOVA to evaluate research question 1, which considered how nationality differences of foreign nurse aides affected work adjustment. The work adjustment scale was used for twenty questions is a six point scale (1 very disagree; 2 disagree; 3 somewhat disagree; 4 somewhat agree; 5 agree; 6 very agree) to avoid respondents to go neutral on the questions. We listed only those that were statistically significant affected work adjustment which are degree of working successfully, rules obedience, feeling it is a right decision to come to Taiwan for work, not planning to go back to one's own country before the contract end, and setting up work goals. The P value of .05 is the border line of acceptable error level [31].

First, regarding the degree of working successfully, the P value is 0.002, and the average of Indonesian nurses is 4.79, which is higher than the 4.60 of Vietnamese nurses and the 3.65 of Filipino nurses. Second, regarding the question of rules obedience, the P value is 0.005. The average for Indonesian nurses is 5.13, which is 0.8 greater than that of Vietnamese nurses (4.33) and 1.01 greater than that of Filipino nurses (4.12). Third, the P value of the right decision to come to Taiwan for work is 0.014; the average of Indonesian nurses is 5.17, which is greater than that of Filipino nurses (4.35). Fourth, the P value of not planning to go back to one's own country before the contract end is 0.014; the average of Indonesian nurses is 5.13, which is greater than that of Filipino nurses (4.18). Fifth, regarding the setting up work goals, the P value is 0.017. The average of Indonesian nurses is 5.25, which is 0.78 higher than that of Filipino nurses (4.47). These results are shown in Table 3.

**Table 3:** ANOVA Test Between Nationality Differences And Work Adjustment

Variable Set		Mean		Significance	Scheffe's Post Hoc Tests
			
	Filipino	Vietnamese	Indonesian		
The Degree Of Working Successfully	3.65	4.60	4.79	0.002	3 > 2, 2 > 1
Rules Obedience	4.12	4.33	5.13	0.005	3 > 1, 3 > 2
The Right Decision To Come To Taiwan For Work	4.35	4.77	5.17	0.014	3 > 1
Not Planning To Go Back To One's Own Country Before The Contract End	4.18	4.37	5.13	0.014	3 > 1
Setting Up Work Goals	4.47	4.83	5.25	0.017	3 > 1

Scheffe's Post Hoc Tests: 1 Filipino; 2 Vietnamese; 3 Indonesian.

In the relationship between work stressors and work adjustment, this study used a bivariate Pearson-product moment correlation analysis to test the association between constructs. In the analysis of correlation, we found that many constructs of work stressors and work adjustment had significant correlations (Table 4).

**Table 4:** The Correlations Between Work Stressors And Work Adjustment

		Task Stressors		Relationship Stressors		System Stressors	
		
		Patient Care Tasks	General Job Tasks	Supervisor	Co-Worker	Workload /Scheduling	Facility Design/Maintenance
**Work Competencies**							
Physical And Mental Skills Applicable To Jobs	*Person Corr*. *Significant*	0.11 0.361	0.151 0.209	-0.254* 0.033	-0.065 0.591	0.137 0.25	-0.024 0.843
Work-Related Interpersonal Skills	*Person Corr*. *Significant*	-0.340** 0.004	0.027 0.822	-0.238* 0.046	-0.025 0.833	-0.257* 0.032	-0.054 0.657
Work Habits	*Person Corr*. *Significant*	0.062 0.61	0.035 0.769	-0.358** 0.003	-0.046 0.701	0.003 0.98	0.245* 0.041
**Work Personality**							
Self-Concept As A Worker	*Person Corr*. *Significant*	0.021 0.861	0.12 0.324	-0.263* 0.028	-0.042 0.726	0.013 0.915	-0.104 0.387
Personal System Of Motivation For Work	*Person Corr*. *Significant*	0.196 0.104	0.063 0.603	-0.067 0.581	-0.033 0.782	0.149 0.218	0.017 0.889
**Work Goals**	*Person Corr*. *Significant*	-0.290* 0.015	-0.023 0.852	-0.266* 0.026	-0.242* 0.044	-0.310** 0.009	0.049 0.692

*P < 0.05, **P < 0.01.

Under the task stressors category, work-related interpersonal skills (-0.34, p < 0.01) and work goals of patient care tasks (-0.29, p < 0.05) had negative correlations with work competency. In the relationship stressors category, the relationship stress from supervisors had a significant negative correlation with work competency (-0.254, p < 0.05; -0.238, p < 0.05; -0.358, p < 0.01), work personality (-0.263, p < 0.05) and work goals (-0.266, p < 0.05). The relationship stress from co-workers had a negative correlation on work goals (-0.242, p < 0.05).

In the system stressors category, the stress from workload/scheduling had a negative correlation with work-related interpersonal skills (-0.257, p < 0.05) and work goals (-0.31, p < 0.01). The facility design/maintenance had a positive correlation with work habit (0.245, p < 0.05). Hence, we derived with two hypotheses: 1) the nationality of foreign nurse aides would have a significant correlation with work adjustment, which has been proven, and 2) work stressors of foreign nurse aides had a significant correlation with work adjustment.

## Discussion

Based on the demographic characteristics of participants, we found that most of the nurses came to Taiwan because of economic pressure placed on them from their families, which is similar to findings from other studies [32,33]. The pressure of raising their children pushed them to work in Taiwan. Most of the foreign nurse aides are well-educated, and their education is higher than that of regular foreign workers.

According to the ANOVA test, Indonesian nurses had greater degrees of successful work than did Vietnamese nurses, who in turn had a greater degree of successful work compared to that of Filipino nurses. This finding is consistent with the Small et al. study [34]. When Indonesian nurses faced work stressors, they had better work adjustment than the other two nationalities, which shows that the difference in the nationality of foreign nurse aides might affect work adjustment. Although most of the foreign nurse aides came from Asian countries, the nationality differences could still have an effect when they were facing work stressors, which contributed to different responses in adapting to new living conditions and cultures. Overall, work stressors had an effect on work, but foreign nurse aides are more likely to adapt to complete their contracts.

Regarding the relationship between work stressors and work adjustment, the stress that was gained from patient care tasks influence the foreign nurse aides' personal relationships at work and also affect their attitude while performing their tasks. Relationships with patients have been found to be a major factor in job satisfaction among nursing aides in other studies [35-37]. Pressure from supervisors could affect the work skills, work habits, personal relationships, self-concepts and work attitudes of the foreign nurse aides [5,23]. A heavy workload and improper scheduling affects the personal relationships and work attitudes of the foreign nurse aides [22,13]. This finding is consistent with that of other studies in which adequate equipment [33] and stable staffing [36,38] were major factors associated with nurse aide job satisfaction, which might lead to longer job tenure.

As a result of our research, we determined four questions that may help improve the working conditions of foreign nurse aides. Would the heavy load of work affect their quality of rest and working abilities? Were there equal returns for their hard working performance? Was the work content assigned reasonable? Were any consolation and encouragement provided to them when they had questions about working regulation? The administrator should take the above questions into consideration. Better design and maintenance of the working environment will allow the foreign nurse aides to have a more efficient work pattern and better performance.

The findings of this study should be considered in light of its limitations. Due to the limitation of manpower, we could only use the long-term care facilities in Tao Yuan County as samples in this study; such a small area for data analysis creates a limitation of the hypothesis. Thus, we cannot present the analysis of foreign nurse aides' work stressors and work adjustments for all of Taiwan.

## Conclusion

From the viewpoint of nationality, work adjustment yielded the most significant difference, which matches our hypothesis. Everyone will encounter stress from the work environment no matter their nationality, their capability to adapt to a new society or culture, or their way of thinking [11-13]. It is important for managers to look at what interventions need to occur to reduce stress among foreign nurse aides.

Our findings indicate that training programs are important factors for work adjustment among foreign nurse aides [25,6]. Previous studies also have shown that an adequate number of well-trained nurses are essential for quality care in long-term care settings [38,8]. Managers can hold regular recognition programs for foreign nurse aides who provide good care for the residents. Long-term care is professional work, and the attitude of supervisors towards foreign nurse aides will affect their quality of care directly [5,23]. Furthermore, celebration and leisure activities could be provided to help nurses release work stress [14].

More effort should be put into improving the working environment for foreign nurse aides in long-term care settings by providing a supportive and enriching work environment. Furthermore, long-term care managers should provide adequate equipment and supplies, such as daily living requirements, alarm systems and lifting equipment [3]. After these issues are solved, we believe the foreign nurse aides will be pleased to work in Taiwan.

## Competing interests

The authors declare that they have no competing interests.

## Authors' contributions

FH performed this study, (re)wrote the draft of the paper, and revised the text. All authors read and approved the final manuscript.

## Pre-publication history

The pre-publication history for this paper can be accessed here:

http://www.biomedcentral.com/1472-6963/11/192/prepub
